# *Streptomyces artemisiae* MCCB 248 isolated from Arctic fjord sediments has unique PKS and NRPS biosynthetic genes and produces potential new anticancer natural products

**DOI:** 10.1007/s13205-017-0610-3

**Published:** 2017-04-11

**Authors:** M. Dhaneesha, C. Benjamin Naman, K. P. Krishnan, Rupesh Kumar Sinha, P. Jayesh, Valsamma Joseph, I. S. Bright Singh, William H. Gerwick, T. P. Sajeevan

**Affiliations:** 1 National Centre for Aquatic Animal Health, Cochin University of Science and Technology, Fine Arts Avenue, Kochi, Kerala 682 016 India; 2 Center for Marine Biotechnology and Biomedicine, Scripps Institution of Oceanography, University of California, La Jolla, San Diego, CA 92093 USA; 3 National Centre for Antarctic and Ocean Research, Headland Sada, Vasco da Gama, Goa 403804 India

**Keywords:** Actinomycetes, *Streptomyces*, Anticancer, Natural products, Apoptosis

## Abstract

After screening marine actinomycetes isolated from sediment samples collected from the Arctic fjord Kongsfjorden for potential anticancer activity, an isolate identified as *Streptomyces artemisiae* MCCB 248 exhibited promising results against the NCI-H460 human lung cancer cell line. H460 cells treated with the ethyl acetate extract of strain MCCB 248 and stained with Hoechst 33342 showed clear signs of apoptosis, including shrinkage of the cell nucleus, DNA fragmentation and chromatin condensation. Further to this treated cells showed indications of early apoptotic cell death, including a significant proportion of Annexin V positive staining and evidence of DNA damage as observed in the TUNEL assay. Amplified PKS 1 and NRPS genes involved in secondary metabolite production showed only 82% similarity to known biosynthetic genes of *Streptomyces*, indicating the likely production of a novel secondary metabolite in this extract. Additionally, chemical dereplication efforts using LC–MS/MS molecular networking suggested the presence of a series of undescribed tetraene polyols. Taken together, these results revealed that this Arctic *S. artemisiae* strain MCCB 248 is a promising candidate for natural products drug discovery and genome mining for potential anticancer agents.

**Electronic supplementary material:**

The online version of this article (doi:10.1007/s13205-017-0610-3) contains supplementary material, which is available to authorized users.

## Introduction

Microorganisms have been the source of many chemotherapeutics used in cancer treatment, and amongst them, actinomycetes are the most promising source organisms. Actinomycetes account for about 45% of all microbial secondary metabolites, of which 7600 (80%) are produced by *Streptomyces* (Bérdy 2005). Over the years, the rate of discovery of new bioactive compounds has reduced, but the rediscovery of known compounds has increased (Fenical et al. 1999). Hence there is a need for bioprospecting of unexplored or underexplored habitats for unique and rare microorganisms, as these can be expected to yield a higher percentage of novel metabolites with desirable bioactivities (Bredholt et al. 2008; Pathom-aree et al. 2006). The Arctic region remains one of the least well-explored geographical locations on Earth for novel bioactive metabolites. Herein, we report on the isolation and screening of actinomycetes from sediment collected from an Arctic fjord and discovery of the production of potential anticancer natural products.

## Materials and methods

### Isolation of actinomycetes

Sediment samples were collected using a van Veen grab from varying depths (50–250 m) in the Arctic Fjord ‘Kongsfjorden’ located in Ny-Ålesund, Svalbard in, July 2012. The samples were serially diluted in ice-cold sterile seawater and plated on Actinomycetes Isolation Agar (AIA) (Himedia, India) supplemented with 0.2 mg/L gentamycin, 0.25 mg/L cycloheximide, and 0.1 mg/L amphotericin B. These were incubated at 20 °C for 4 weeks at which time characteristic actinobacterial colonies were isolated and their extracts screened for anticancer activity as described below.

### Fermentation and crude extract preparation

The isolated strains of actinomycetes were mass cultured for the production of their secondary metabolites. Briefly, a loop full of actinomycete spores was inoculated into a seawater-based seed medium (beef extract 3 g/L, peptone 5 g/L). The flasks were incubated at 20 °C for 48 h on a rotary shaker set at 150 rpm. Subsequently, these were transferred to seawater-based fermentation medium (Yang et al. 2013) (soybean meal 3 g/L, yeast extract 3 g/L, proline 1 g/L, beef extract 3 g/L, glycerol 6 mL/L, K_2_HPO_4_ 0.5 g/L, MgSO_4_·7H_2_O 0.5 g/L, FeSO_4_·7H_2_O 0.5 g/L, CaCO_3_ 2 g/L, pH 7.4) and incubated at 20 °C for 10 days on a rotary shaker at 150 rpm. The liquid cultures were centrifuged at 6500 g for 5 min, and the supernatant was extracted three times with an equal volume of ethyl acetate. The extract was concentrated under reduced vacuum on a rotary evaporator at 40 °C, and the dry residue (~10 mg) was re-dissolved in 1 mL DMSO for cytotoxicity screening against NCI-H460 non-small cell human lung cancer (NSCLC) cells as well as for chemical dereplication efforts.

### Screening for potential anticancer activity using NCI-H460 cells in vitro

Cytotoxicity evaluation of the crude extracts was performed using a sulforhodamine B (SRB) (Sigma, USA) colorimetric assay on 96-well culture plates (Skehan et al. 1990). The NCI-H460 cell line, obtained from the National Centre for Cell Science (NCCS, Pune, India), was maintained in RPMI-1640 (Himedia, India) supplemented with 10% fetal bovine serum (FBS) (Himedia, India) at 37 °C in an incubator with 5% carbon dioxide. Aliquots of 190 µL cell suspension at a density of 1.9 × 10^4^ cells/well were pipetted into 96-well micro titer plates. The crude extracts were diluted to 1 mg/mL with sterile deionized water, and then 10 µL of each were added to each well to achieve a final concentration of 50 µg/mL. Control wells were composed of 190 µL cell suspension plus 10 µL of 10% DMSO. All assays were performed in triplicate. The plates were incubated at 37 °C in a CO_2_ (5%) incubator for 72 h, and fixed with 100 µL ice-cold 30% trichloroacetic acid (TCA) and incubated at 4 °C for another 1 h. The plates were gently washed four times and air-dried at room temperature. To each well, 100 µL of 0.057% (wt/vol) sulforhodamine B (SRB) prepared in deionized water with 1% acetic acid was added to each well and incubated at room temperature for 30 min. Unbound stain was removed by washing with 1% acetic acid and then the plates were air dried. To dissolve the cell bound dye, 200 µL of 10 mM Tri base solution (pH 10.5) was added to each well and the plate was shaken on a gyratory shaker for 10 min. Optical density (OD) was read at 510 nm in a microplate reader (Tecan, Switzerland). Percentage of cell-growth inhibition (GI) was calculated according to the following equation (Vichai and Kirtikara 2006):

$$ {\text{Percentage}}\;\;{\text{of}}\;\;{\text{growth}}\;\;{\text{inhibition}} = 100 - {\text{percentage of control cell growth,}} $$

$$ {\text{Percentage}}\;{\text{of}}\;{\text{control}}\;{\text{cell}}\;{\text{growth}} = ({\text{mean}}\;{\text{OD}}\;{\text{sample}} - {\text{mean}}\;{\text{OD}}\;{\text{day}}\; 0)/({\text{mean}}\;\;{\text{OD}}\;\;{\text{negative}}\;{\text{control}} - {\text{mean}}\;{\text{OD}}\;{\text{day}}\; 0) \times 100. $$

Based on the results of a primary screening effort, a potent actinomycete isolate, MCCB 248, was selected for further studies. IC_50_ values were determined to the NCI-H460 human lung cancer cell line as well as to a cell line derived from normal epithelial kidney cells from the African green monkey (BS-C-1). Briefly, two fold serial dilution of the crude extract of the MCCB 248 isolate was prepared to obtain concentrations ranging from 1 mg/mL to 15.625 µg/mL in 10% DMSO; these were added to the various cell lines as described above. IC_50_ values were determined based on probit analysis (Brownlee et al. 1952).

### Molecular identification of MCCB 248 by 16S rRNA Gene Analysis

The 16S rRNA gene from isolate MCCB 248 was PCR-amplified, sequenced, and a phylogenetic tree was constructed. Briefly, DNA was extracted from a 48 h broth culture of strain MCCB using the PureLink Genomic DNA mini kit (Invitrogen, USA). PCR amplification of the 16S rRNA was performed using universal primers 16S1 (GAGTTTGATCCTGGCTCA) and 16S2 (ACGGCTACCTTGTTACGACTT). The amplified PCR product was purified by Exosap (Affymetrix, USA) and sequenced. Nucleotide sequence data were analyzed using BLAST and deposited in NCBI GenBank. The nucleotide sequences were aligned using Clustal W on MEGA 6 (Tamura et al. 2013). The 16S rRNA sequences of closely related actinomycetes were retrieved from GenBank, and their similarity to the present isolate was assessed at the nucleotide level. A phylogenetic tree was constructed using the neighbor-joining method with boot strap values based on 1000 replicates.

### Amplification of biosynthetic genes encoding for secondary metabolites

PCR based screening for polyketide synthases (PKS I & PKS II), nonribosomal peptide synthetases (NRPS), aminodeoxyisochorismate synthase (phzE), and cytochrome P450 hydroxylase (CYP) genes was performed using the degenerate primers reported previously (Izumikawa 2003; Wawrik et al. 2005; Ayuso-Sacido and Genilloud 2005; Lee et al. 2006; Schneemann et al. 2011). The composition of the reaction mixture for all PCR amplifications was: 0.4 µL template, 5 µL 2× EmeraldAmp GT PCR Master Mix (Takara Bio Inc., Japan), and 0.5 µL each of the primers (10×).

PCR conditions were as described below.

PKS 1 (10 µL): 2 min at 95 °C, followed by 25 cycles of 40 s at 95 °C, 40 s at 62 °C and 45 s at 72 °C, followed by a 5 min extension at 72 °C.

PKS 2 (10 µL): 5 min at 95 °C, followed by 40 cycles of 1 min at 95 °C, 30 s at 62 °C and 1 min at 72 °C, followed by a 10 min extension at 72 °C.

NRPS (10 µL): 5 min at 95 °C, followed by 35 cycles of 30 s at 95 °C, 2 min at 59 °C and 4 min at 72 °C, followed by a 10-min extension at 72 °C (Yuan et al. 2014).

phzE (10µL): 2 min at 94 °C, followed by 36 cycles of 1 min at 94 °C, 1 min at 54.7 °C and 2 min at 72 °C, followed by a 7-min extension at 72 °C (Yuan et al. 2014).

CYP (10µL): 5 min at 96 °C, followed by 45 cycles of 1 min at 96 °C, 30 s at 60 °C and 45 s at 72 °C, followed by a 5-min extension at 72 °C (Yuan et al. 2014).

Similarity searching for the gene sequences was performed using BLASTX against the NCBI GenBank. For phylogenetic analysis, the nucleic acid sequences were translated to protein sequences and aligned with other similar biosynthetic proteins in the NCBI database using the Clustal W program on MEGA 6 (Tamura et al. 2013). A phylogenetic tree of the corresponding biosynthetic genes was constructed by the neighbor-joining method with boot strap values based on 1000 replications.

### Chemical dereplication efforts by LC-PDA-MS/MS and molecular networking

An aliquot (1 mg) of the fermentation culture extract was prepared for chemical analysis by filtration over GracePure C18-Max 100 mg/1 mL SPE cartridges (Grace Technologies, USA). Compounds were eluted from the column using acetonitrile and methanol to produce a final volume of 1 mL. This material was taken for nominal mass resolution LC-PDA-MS/MS analysis on a Thermo Finnigan system with a Surveyor PDA Plus Detector, Autosampler Plus, and LC Pump Plus coupled to an LCQ Advantage Plus mass spectrometer (Thermo Fisher Scientific, USA), and with a Phenomenex Kinetex 150 mm × 10 mm × 5 µm C_18_ analytical column installed (Phenomenex, USA). The UV–Vis spectrum from 200 to 600 nm and positive mode ESI mass spectrum from *m*/*z* 190–2000 were recorded, and the mass spectrometer was configured for an automated sample-dependent MS/MS scan. Data were analyzed both manually and by mass spectrometric molecular networking using the Global Natural Products Social Molecular Networking (GNPS) (Wang et al. 2016). This was performed in duplicate using both the native library searching and analog detection search modes. Additionally, for compound dereplication masses of major components of the mixture were searched manually using MarinLit and the Dictionary of Natural Products databases.

### Evaluation of apoptosis by Hoechst 33342 staining

To evaluate morphological changes induced by the *Streptomyces sp.* MCCB 248 metabolites, NCI-H460 cells were stained with Hoechst 33342 and observed for characteristics of apoptosis using a fluorescence microscope (Zhang et al. 2007). Briefly, NCI-H460 cells, seeded in a chamber slide having 1.9 × 10^4^ cells per well, were treated with the culture extract at its IC_50_ value. After 24 h of treatment, the culture medium was removed and cells were washed twice with PBS and stained with DNA specific Hoechst 33342 dye (Sigma Chemicals, USA) (2 μg/mL in PBS) for 10 min at 37 °C. At the end of staining, cells were observed under a fluorescence microscope for apoptotic features. Doxorubicin (Sigma, USA) was used as a positive control and DMSO was used as a negative control.

### Evaluation of apoptosis by terminal deoxynucleotidyl transferase dUTP nick end labeling (TUNEL) assay

To detect cells undergoing apoptosis, in situ labeling of the 3′ OH end of the DNA fragments generated during apoptosis was performed using the In Situ Cell Death Detection Kit (TUNEL) (Roche Diagnostics, Switzerland) according to the manufacturer’s instructions. Briefly, NCI-H460 cells in their log phase were treated with the culture extract at its IC_50_ value and incubated for 24 h. Subsequently, culture medium was removed and cells were washed with PBS, air-dried and fixed with 4% paraformaldehyde (in PBS) for 1 h at 25 °C. Fixed cells were washed 3 times with PBS and incubated with permeabilization solution (1% Triton-X in 1% sodium citrate) for 2 min at 4 °C. As a positive control, NCI-H460 cells were fixed, permeabilized and treated with recombinant DNA*ase* I (New England Biolabs, USA) for 10 min to induce DNA strand nicks. Both control and treated cells were washed twice with PBS, and then 50 µL of TUNEL reaction mixture was added to each well. The cells were incubated for 1 h at 37 °C in a humidified dark chamber and subsequently washed three times with PBS and observed under a fluorescence microscope.

### Annexin V–propidium iodide (PI) double staining

Externalization of membrane phosphatidylserine (PS) is an early event occurring in cells undergoing apoptosis, and can be visualized with FITC labeled Annexin V staining. Annexin V is a Ca^2+^ dependent phospholipid-binding protein that has high affinity for PS, and binds to cells with exposed PS. To confirm apoptosis induction by *Streptomyces* sp. MCCB 248 metabolites, Annexin-V-FLOUS/Propidium iodide (PI) staining was performed as per the manufacturer’s instructions (Roche Diagnostics, Switzerland). Briefly, cells grown in chamber slides (Millicell EZ slide, Millipore Corporation, US) were treated with the MCCB 248 extract at its IC_50_ value. After adding the extract, cells were observed and counted under a fluorescent microscope for apoptotic features at regular intervals of treatment (0, 6, 12 and 24 h). A minimum of four different microscopic fields was counted and the average calculated and expressed as a percentage of the total cell population. Cells treated with 10% DMSO were used as a negative control.

## Results

### Isolation of actinomycetes and screening for anticancer activity

**Fig. 1:**
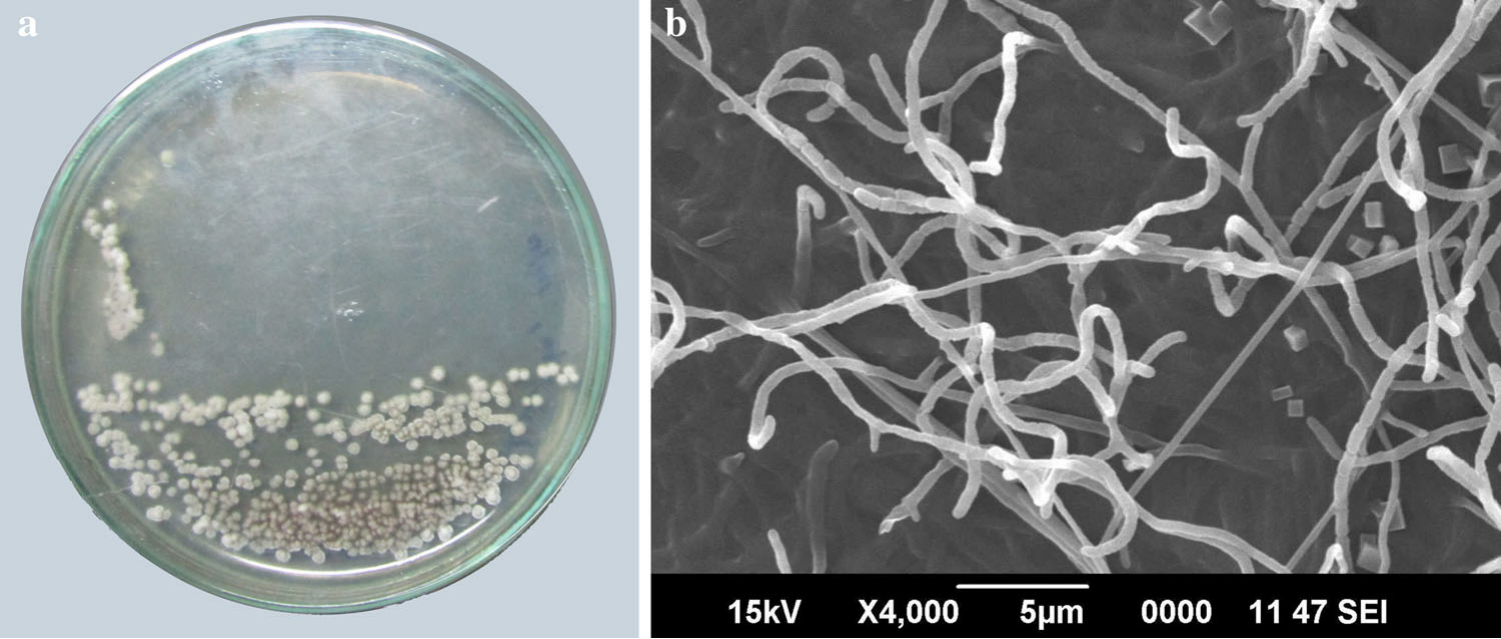
**a** Colony morphology and **b** scanning electron micrograph of aerial mycelia of *S. artemisia* strain MCCB 248 after incubation for 14 days on Nutrient agar at 28 °C

**Fig. 2:**
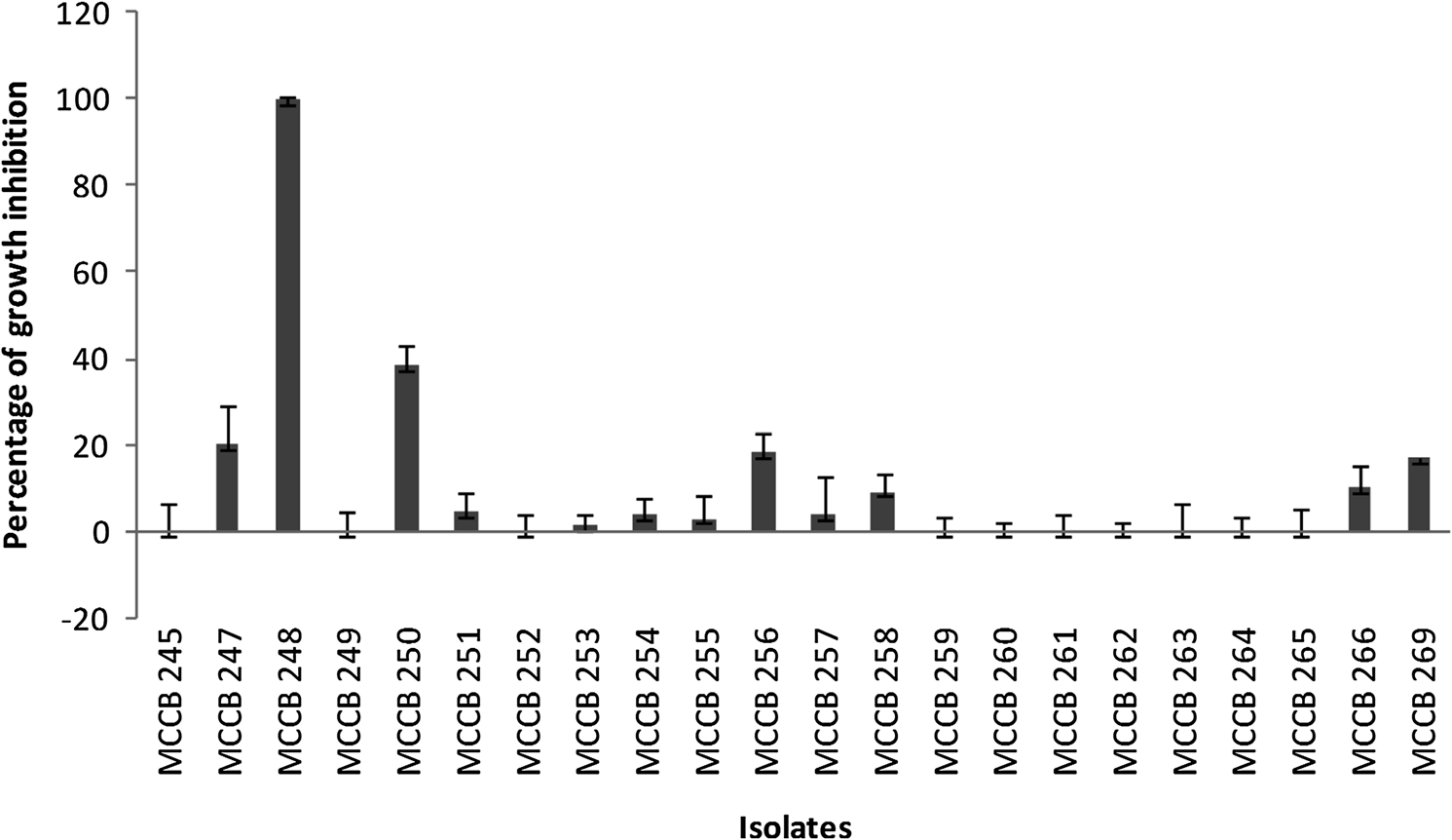
Percentage growth inhibition by various actinomycete crude extracts on NCI-H460 cell lines at 50 µg/mL (normalized to a control without extract)

**Fig. 3:**
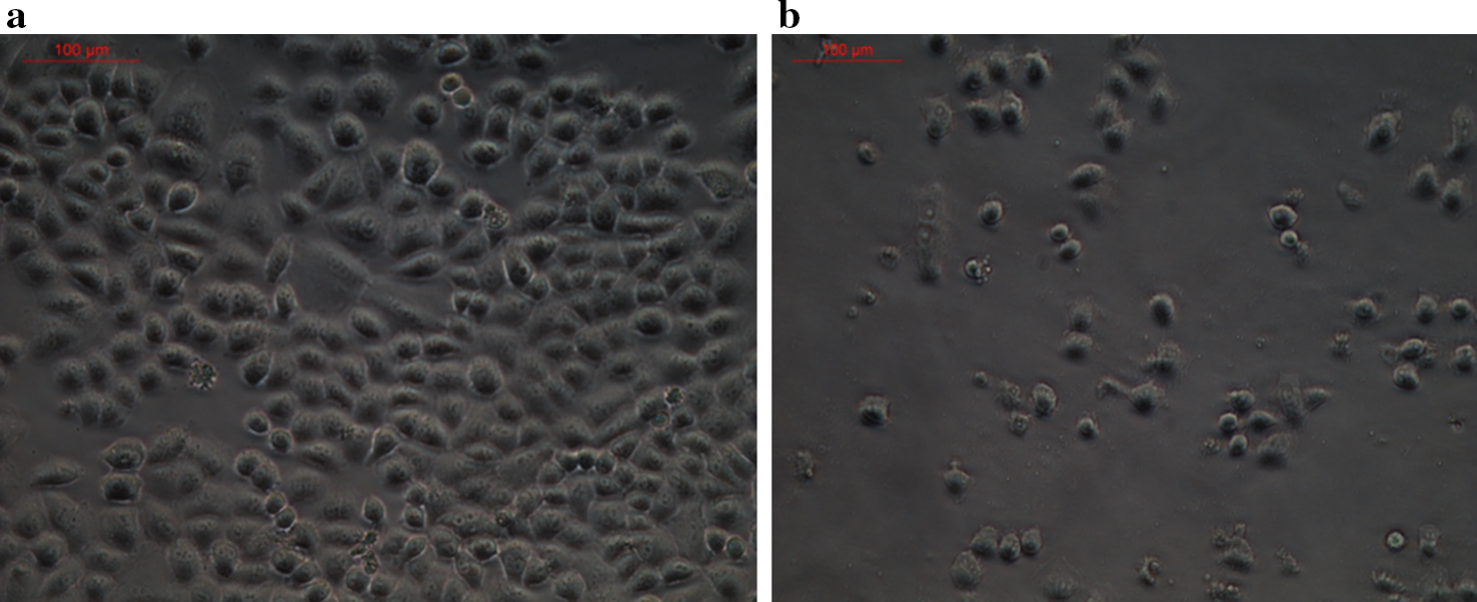
Phase contrast microscope image (20×) of NCI-H460 cells; **a** control cells, **b** treated cells with *S. artemisiae* MCCB 248 extract at its IC_50_ value for 24 h

A total of 22 morphologically distinct actinobacterial isolates were obtained from the Arctic sediment samples. Preliminary screening for anticancer activity of the extracts of these isolates resulted in the identification of one isolate, designated as MCCB 248 (Fig. 1), as possessing the most potent growth inhibition against NCI-H460 cells (Fig. 2). Upon further study, the IC_50_ of the MCCB 248 crude extract was found to be 8.1 and 8.2 µg/mL in NCI-H460 human non-small cell lung cancer cells and non-cancerous BS-C-1 African green monkey kidney cells, respectively. During this preliminary screening effort, it was observed that cells exposed to this extract were less confluent after 24 h of incubation, and attached to the substratum with pronounced shrinkage (Fig. 3).

### Molecular identification of the isolate MCCB 248

**Fig. 4:**
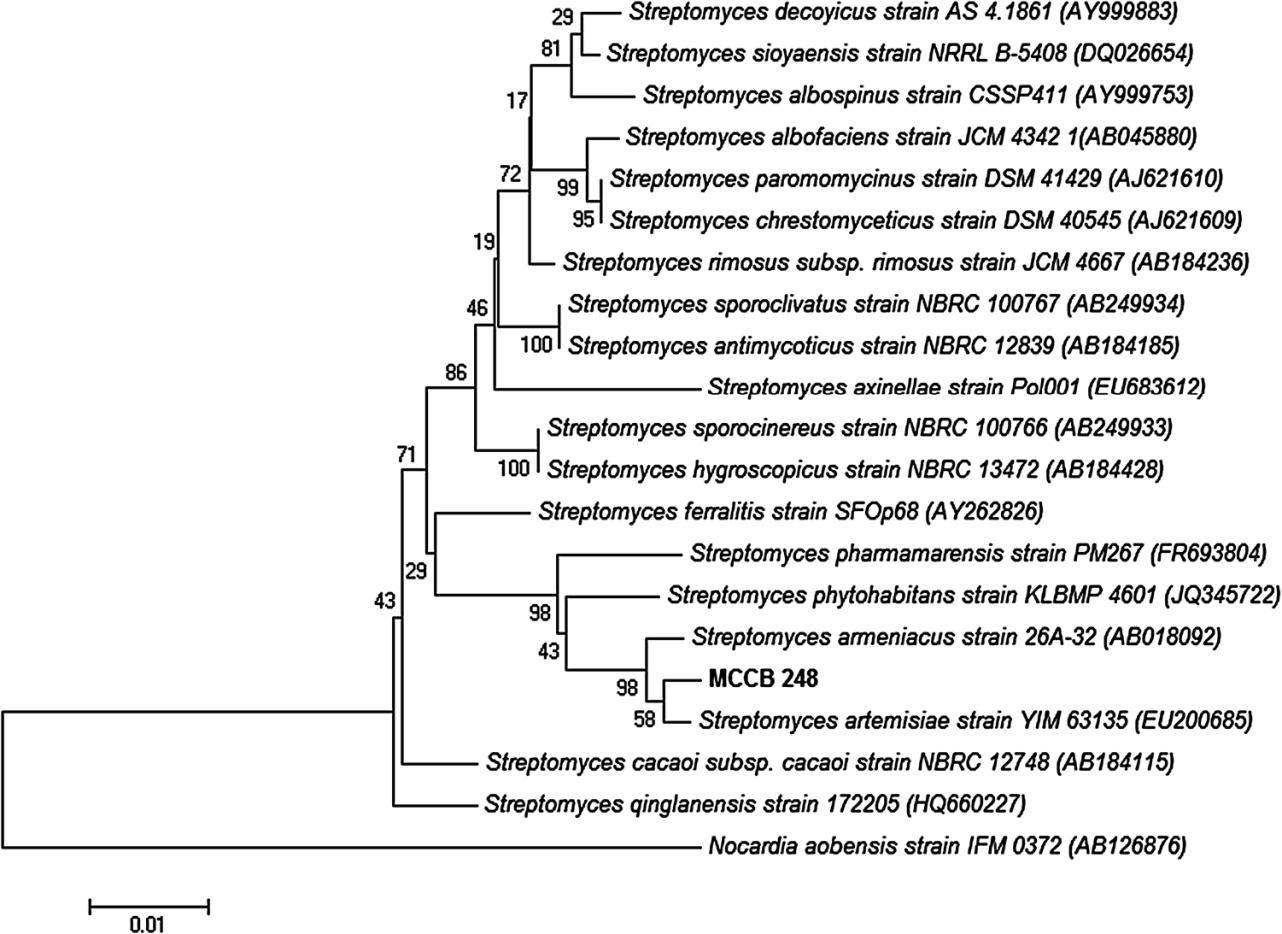
Neighbour-joining phylogenetic dendrogram based on 16S rRNA gene sequences showing relationships between the isolated *S. artemisiae* MCCB 248 and related taxa

A 16S rRNA gene sequence consisting of 1350 bp from the isolate MCCB248 identified it by BLAST analysis as belonging to the genus *Streptomyces*, having highest sequence similarity (99%) to the type strain *Streptomyces artemisiae* YIM 63135. The latter strain was isolated from surface-sterilized tissue of *Artemisia annua* L., collected in Yunnan Province, southwest China,and described by (Zhao et al. 2010). Moreover, phylogenetic analysis using the neighbor-joining method positioned it in a distinct clade along with *Streptomyces artemisiae* YIM 63135 (Fig. 4). Accordingly, the isolate MCCB 248 was identified as *Streptomyces artemisiae,* and hence designated as *Streptomyces artemisiae* MCCB 248 (GenBank accession number KP313874).

### Detection and phylogenetic analysis of biosynthetic genes in *Streptomyces artemisiae* MCCB248

After screening for the presence of genes involved in the biosynsthetic pathways, such as type I polyketide synthase (PKS I), type II polyketide synthase (PKS II), nonribosomal peptide synthetase (NRPS), aminodeoxyisochorismate synthase (phzE), and cytochrome P450 hydroxylase (CYP), *Streptomyces artemisiae* MCCB 248 was found to have type I polyketide synthase (PKS I) (GenBank accession number KT251042) and nonribosomal peptide synthase (NRPS) (KT277491) genes. After NCBI GenBank BLASTX searching, the PKS 1 nucleotide sequence had its closest match with *Streptomyces* sp. ID05-A0179 (83%). Phylogenetic analysis of the *S. artemisiae* MCCB248 PKS 1 using translated nucleic acid sequences revealed that it formed a separate cluster along with *Streptomyces himastatinicus* ATCC 53653 and *Streptomyces aurantiacus* JA4570 (Supplementary material). Blast analysis of the NRPS sequence from *S. artemisiae* MCCB248 showed that it had closest similarity (83%) with one from *Streptomyces* sp. SCAU5132. Phylogenetic analysis of this NRPS using the deduced amino acid sequence positioned *S. artemisiae* MCCB 248 as a distinct clade in the tree (Supplementary material).

### Chemical dereplication efforts by LC-PDA-MS/MS and molecular networking

The crude extract of *S. artemisiae* MCCB 248 was first subjected to a C_18_ SPE clean-up step, and then analyzed by positive ionization LC–MS/MS analysis. Four major chemical components were determined to be present in the crude extract. These four molecules had mass spectra indicating protonated mass ions of *m/z* 761, 827, 879, and 913, and appeared to possess a common UV chromophore (*ν* = 312 nm, broad absorption from 280 to 340 nm, and *ν* = 240 nm). These MS/MS spectra did not match with any library entry upon molecular networking. However, they clustered together in a small molecular family, and upon library comparison with the analog search option, were suggested to be structurally related to a polyhydroxy macrolide, such as bastimolide A (Shao et al. 2015). A number of minor metabolites were also detected in the chromatogram by MS or DAD UV, but were not analyzed further due to their low abundance.

### DNA damage and induced apoptosis

**Fig. 5:**
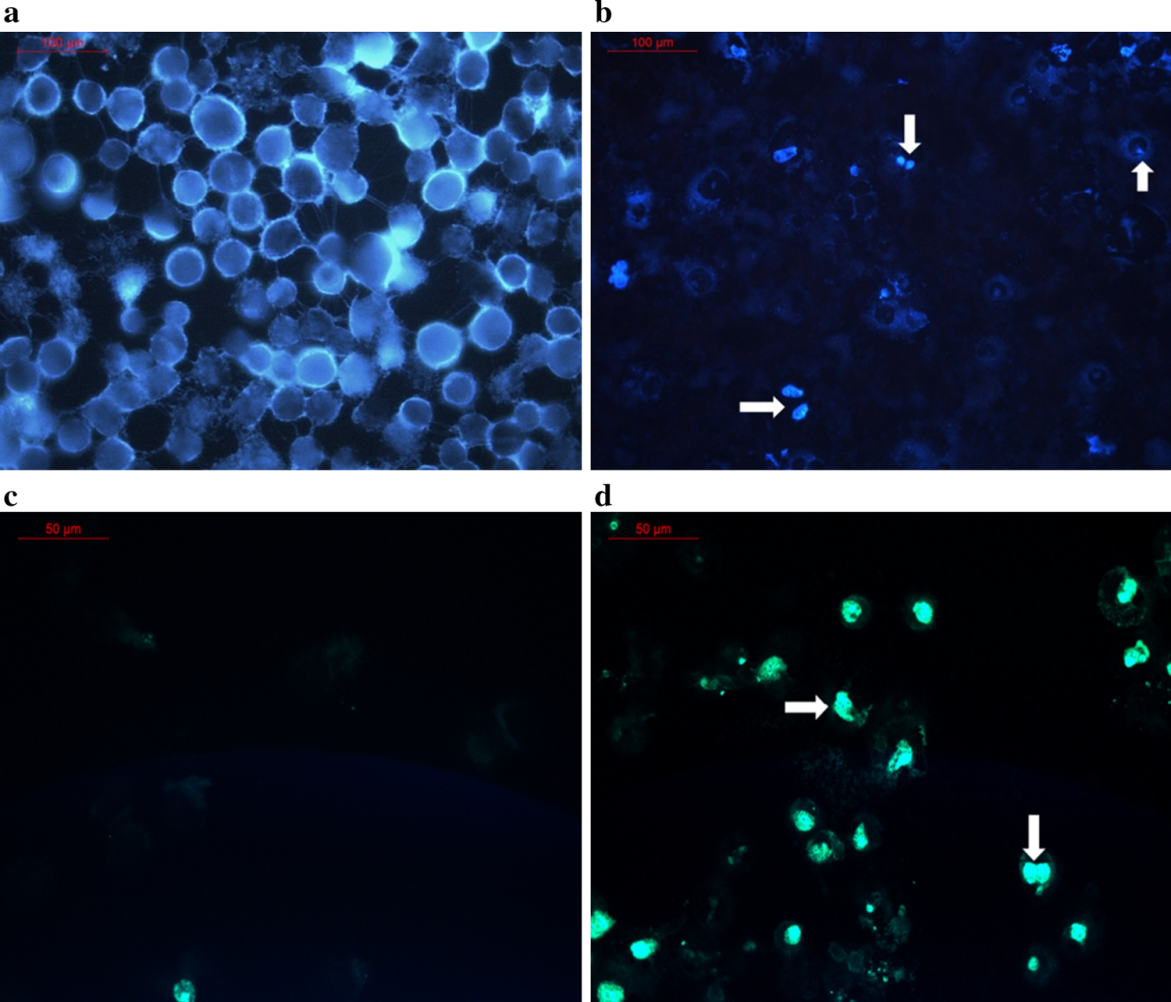
Fluorescent microscope image (20×) of Hoechst 33342 stained H-460 cells; **a** control cells, and **b** *S. artemisiae* MCCB 248 extract treated cells. Also shown are fluorescent microscope images (40×) of the TUNEL assay of **c** control cells, and **d** *S. artemisiae* MCCB 248 extract treated cells for 24 h

NCI-H460 cells exposed to the *S. artemisiae* MCCB248 extract exhibited a characteristic apoptotic morphology, such as shrinkage of cell nuclei, chromatin condensation and nuclear fragmentation (Fig. 5a, b), which indicated that one or more components of this extract induced apoptosis. This was further in evidence using a TUNEL assay, which demonstrated condensed TUNEL positive chromatin within the cell nuclei (Fig. 5c, d) when treated with the *S. artemisiae* MCCB 248 extract. DNase I treated cells were used as a positive control.

**Fig. 6:**
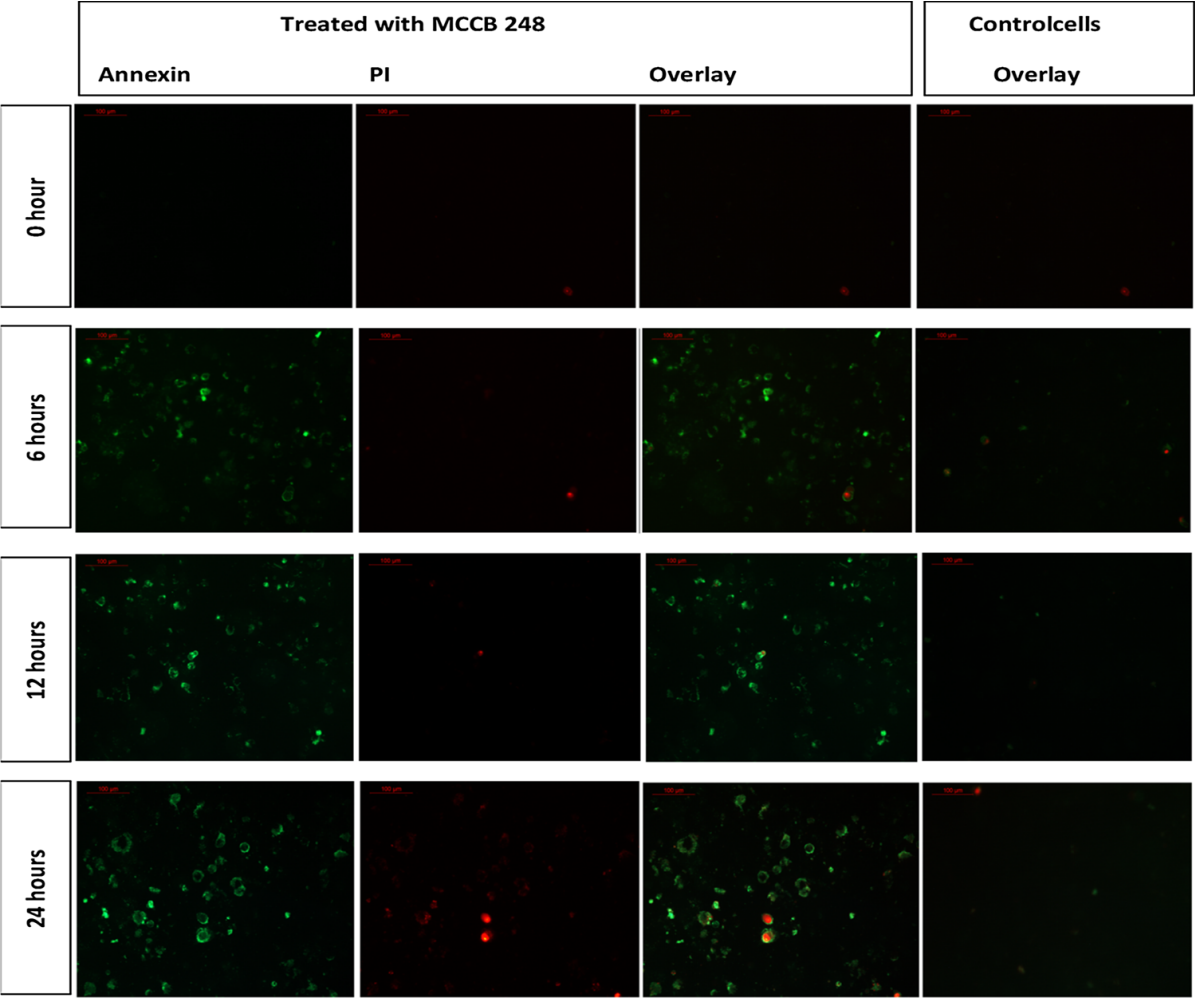
Fluorescent microscope image (20×) of Annexin-V/PI double-staining assay. After treating with *S. artemisiae* MCCB 248 extract, cells were stained with Annexin V-FITC and propidium iodide and analyzed after 0, 6, 12 and 24 h of treatment

**Fig. 7:**
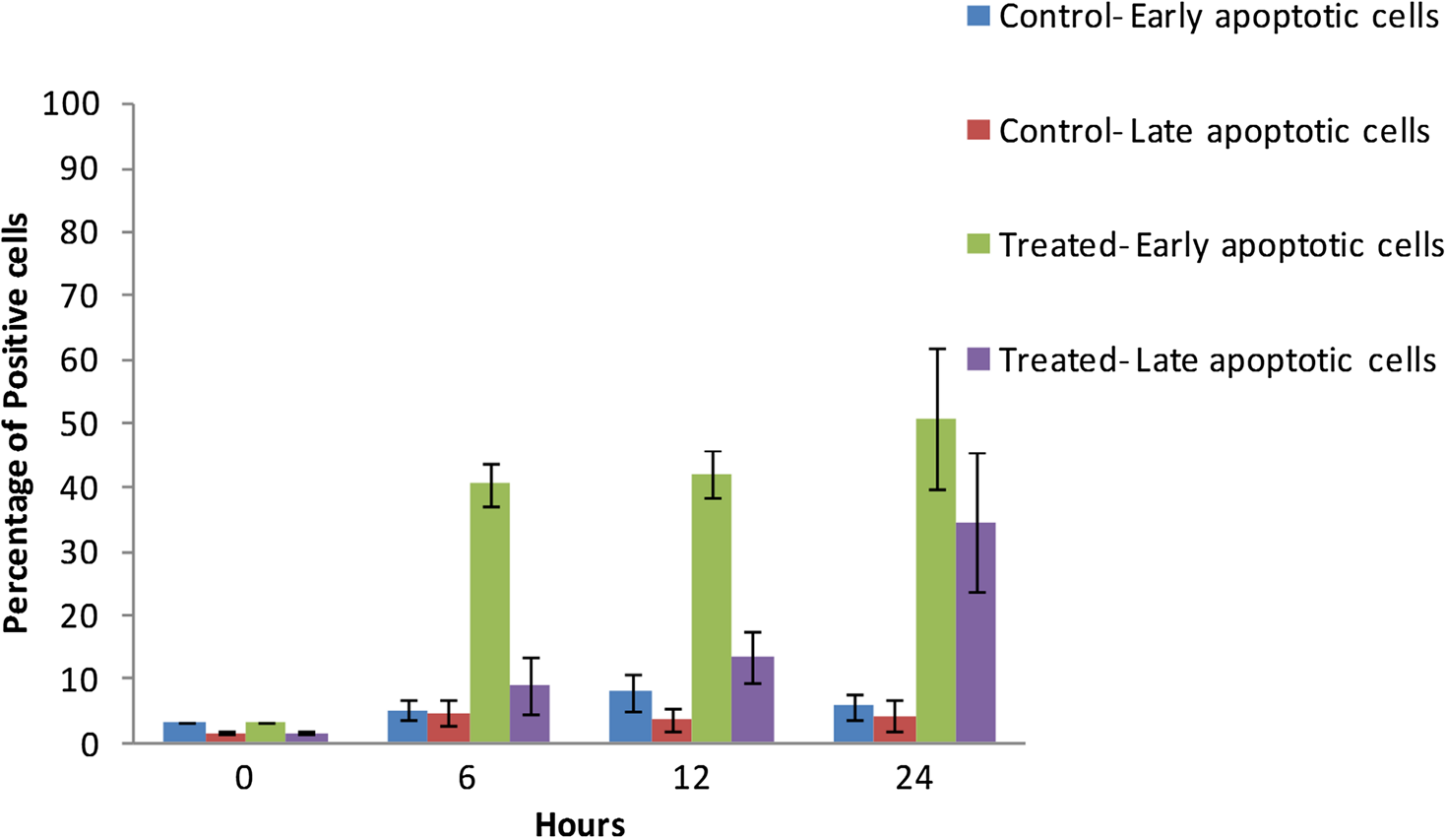
Percentage of H-460 cells showing early and late apoptotic cell death at 0, 6, 12, 24 h after exposure to the extract of *S. artemisiae* MCCB 248 (data shown are mean of four independent observations and its standard deviation)

In Annexin V-PI assays, a time-dependent increase in Annexin V positive cells was observed in cells treated with *S. artemisiae* MCCB 248 extract, implying that there was an increased PS translocation to the outer leaflet of the plasma membrane. At the beginning of treatment (0 h), a majority of the cells were negative for both Annexin V and propidium iodide **(**PI) staining. After 6 h of treatment, 40% of the cells were Annexin V positive and only 9% of cells were both Annexin V and PI positive. A similar observation was observed after 12 h (42% Annexin V and 13% both Annexin V and PI positive reaction), which was indicative of the early stages of apoptosis. After 24 h, 51% of cells were Annexin V positive and more cells had become positive for PI (34%), indicative of the later stages of apoptosis and signified more dead cells (Figs. 6, 7).

## Discussion

Actinomycetales are excellent sources of novel bioactive metabolites with potential pharmaceutical applications (Lam 2006). Actinomycetes contribute over half of the known bioactive secondary metabolites, especially in the classes of antibiotics, antitumor agents, enzymes and immunosuppressive agents (Bérdy 2005). With improved sampling and culture techniques, the isolation of new groups of actinomycetes are being made from sediments collected from even the deepest parts of the oceans (Kwon et al. 2006; Pathom-aree et al. 2006). Such efforts are yielding such chemically rich genera as *Salinispora* (Feling et al. 2003) and *Marinispora* (Kwon et al. 2006).

In the present work, 22 different actinomycetes were isolated and cultured from sediment samples collected from the Arctic fjord, Kongsfjorden. Among these was *S. artemisiae* MCCB248, the extract of which exhibited cytotoxicity with IC_50_ values of 8.1 µg/mL on NCI-H460 cells and 8.2 µg/mL on BS-C-1 cells. The biosynthetic potential of this microorganism for the production of secondary metabolites was evaluated by detecting the presence of genes involved in secondary metabolite production. The presence of PKS 1 and NRPS genes in *S. artemisiae* MCCB 248 suggests the possibility that it can produce bioactive secondary metabolites belonging to these two classes of natural products, or a hybrid of both. Relatively low sequence similarity of the PKS and NRPS gene sequences with those available in GenBank indicates the possibility that novel compounds are produced by *S. artemisiae* MCCB 248. By phylogenetic analysis of PKS 1 using the deduced amino acid sequence, it formed a separate branch along with *Streptomyces himastatinicus ATCC 53653* and *Streptomyces aurantiacus.*

In the present study, H460 human non-small cell lung cancer cells treated with the *Streptomyces* sp. MCCB 248 extract showed nuclear condensation and fragmentation as evident from Hoechst 33342 staining. This is similar to what is observed with the known anticancer agent doxorubicin (Xin et al. 2012; Zhang et al. 2010). A significant number of anticancer agents display cytotoxicity by damaging DNA, leading to apoptosis (Johnstone et al. 2002). The morphological hallmark of apoptotic cell death includes shrinkage of the cell and nucleus as well as condensation of nuclear chromatin (Saraste 2000), as was observed with application of the *S. artemisiae* MCCB248 extract.

The DNA fragmentation observed in the TUNEL assay confirmed apoptotic cell death in cells treated with the *S. artemisiae* MCCB248 extract. Further, a time-dependent increase in Annexin V positive cells implied more cells had undergone a flip-flop of PS to the outer leaflet of the plasma membrane, another indicator of apoptosis. These results demonstrated that *S. artemisiae* MCCB 248 extract induced apoptotic cell death in NCI-H460 lung cancer cells.

The HPLC–PDA-MS/MS chemical analysis and dereplication efforts for the crude extract of *S. artemisiae* MCCB 248 suggested some key molecular features associated with the major components of this mixture. The MS and MS/MS spectra of the major four metabolites in the extract showed repeated losses of 18 mass units, which is typical of polyols that show neutral ion losses of water. Furthermore, the UV–Vis spectrum associated with these compounds was suggestive of a tetraene moiety in each, due to the typical absorption band from 280 to 340 nm with a clear maxima at 312 nm (Supplementary material). Taken together, these data suggest the presence of tetraene polyols in the extract, which would be in-line with the production of PKS or hybrid PKS natural products by similar actinomycetes such as the novonestmycins, separacenes, bahamaolides and marinisporolides (Kwon et al. 2009; Kim et al. 2012; Bae et al. 2013; Wan et al. 2015). However, the parent masses observed in this study were not found to correlate with any known molecules of this class in the MarinLit or Dictionary of Natural Products databases. Additionally, the molecular networking performed using GNPS failed to yield any matches against spectra present in the available MS/MS library databases for the four major metabolites present in the extract, but did suggest that these compounds could be analogs of the reported polyhydroxy macrolide natural product known as bastimolide A (Shao et al. 2015). On the other hand, of the lower abundance molecules in the extract, it was possible to identify several known diketopiperazines.

In conclusion, we have isolated and identified the actinomycete *Streptomyces artemisiae* MCCB 248 from an Arctic fjord that showed promising cancer cell toxicity to NCI-H460 cells in vitro. From the cell-based assays, it could be concluded that this activity likely resulted from the induction of apoptotic pathways leading to cell death. Chemical analysis, dereplication efforts and biosynthetic gene comparisons for the isolate suggested that this organism produces potentially novel bioactive secondary metabolites. Such a promising finding warrants further study, including the purification and characterization of the active compounds as well as description of their biosynthetic gene cluster(s).

## Electronic supplementary material

Below is the link to the electronic supplementary material.

Supplementary material 1 (DOCX 32 kb)

Supplementary material 2 (DOCX 412 kb)
