# Supplementary material for: Streptomyces artemisiae MCCB 248 isolated from Arctic fjord sediments has unique PKS and NRPS biosynthetic genes and produces potential new anticancer natural products
Source: 3 Biotech. 2017 Apr 11;7(1):32. doi: 10.1007/s13205-017-0610-3 (PMC5388659; doi:10.1007/s13205-017-0610-3)

**Supplementary material 1**

Representative neighbour-joining phylogenetic tree of PKS amino acid sequences


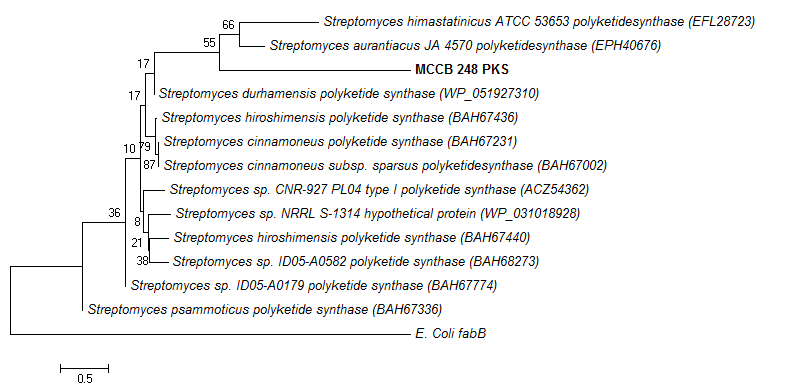


Representative neighbour-joining phylogenetic tree of NRPS amino acid sequences


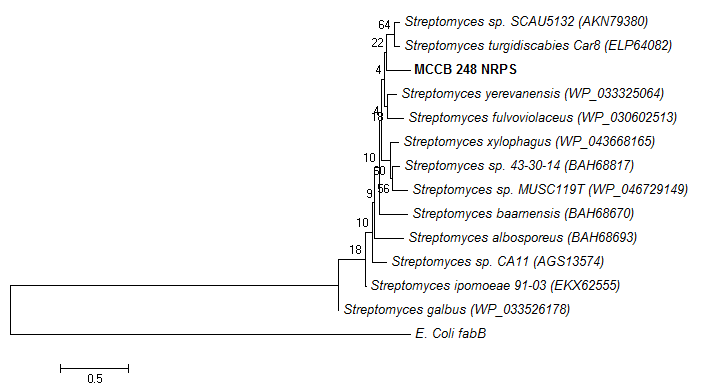

Supplement: Supplementary file 1 — Supplementary material 1 (DOCX 32 kb) [file 13205_2017_610_MOESM1_ESM.docx]
