# Supplementary material for: Streptomyces artemisiae MCCB 248 isolated from Arctic fjord sediments has unique PKS and NRPS biosynthetic genes and produces potential new anticancer natural products
Source: 3 Biotech. 2017 Apr 11;7(1):32. doi: 10.1007/s13205-017-0610-3 (PMC5388659; doi:10.1007/s13205-017-0610-3)

**Supplementary material**

LC-PDA-MS chromatogram of *Streptomyces artemisiae* MCCB 248 crude extract.
Instrumental configuration was described in the main text; gradient solvent system follows 0.6 mL/min of 30% acetonitrile: 70% water with 0.1% v/v formic acid for 5 min, increased linearly
to 99% acetonitrile in 17 min, held at 99% acetonitrile for 5 min, returned to 30% acetonitrile in
1 min. Labels denote retention time (above) and maxima peak (below). Peaks of interest were observed from 14-16 min.

LC-MS/MS based molecular network of *Streptomyces artemisiae* MCCB 248 crude extract.
This network was generated using GNPS software with standard settings selected except for minimum matched fragment ions = 2, minimum cluster size = 1, and main pairs cos = 0.5. Nodes have been labeled with their MS parent ion, scaled to size according to the intensity of their MS peak integration, and many self-loop nodes (single peak clusters) were abridged here for brevity.


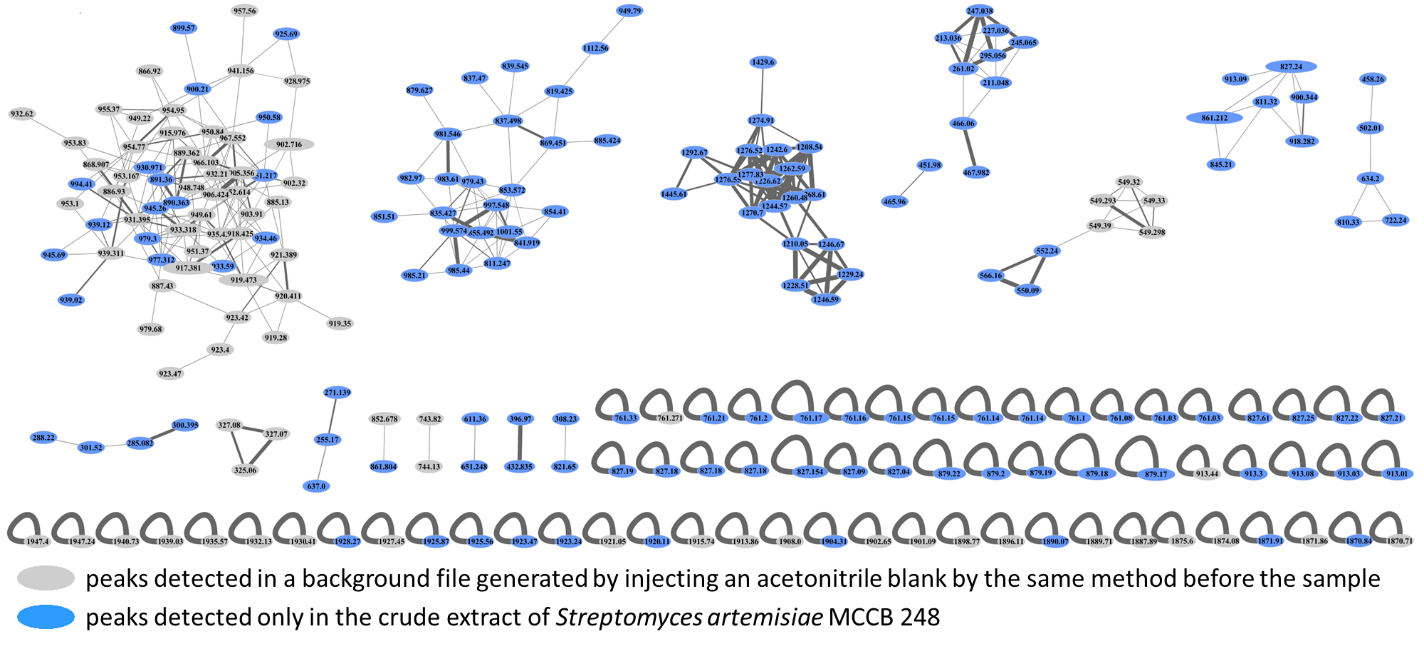

Supplement: Supplementary file 2 — Supplementary material 2 (DOCX 412 kb) [file 13205_2017_610_MOESM2_ESM.docx]
